# A Retrospective Evaluation of Challenges in Urethral Stricture Management in a Tertiary Care Centre of a Poor Resource Community

**DOI:** 10.5812/numonthly.13053

**Published:** 2013-11-13

**Authors:** Abimbola Olaniyi Olajide, Folakemi Olajumoke Olajide, Oladapo Adedayo Kolawole, Ismaila Oseni, Adewale Idowu Ajayi

**Affiliations:** 1 Department of Surgery, Ladoke Akintola University of Technology, Ogbomoso, Nigeria; 2 Department of Community Medicine, Obafemi Awolowo University, Ile-Ife, Nigeria; 3 Department of Radiology, Ladoke Akintola University of Technology, Ogbomoso, Nigeria

**Keywords:** Constriction, Surgical Flaps, Transplants, Inflammation, Fibrosis, Fistula

## Abstract

**Background:**

Management of urethral stricture has evolved over the years with better understanding of the pathology, advancement in imaging, and introduction of several techniques of urethral reconstruction. In sub-Saharan Africa, advancement in management of urethral stricture may not be comparable with what obtained in most developed nations because of problems like late presentation and persistence of rare complications still reported in recent literature from the region.

**Objectives:**

We set to evaluate the challenges faced by urologists involved in the management of urethral strictures in Osogbo, a poor resource community in south western Nigeria.

**Patients and Methods:**

A retrospective study was performed in the urology unit of Ladoke Akintola University of Technology Teaching Hospital, Osogbo, Nigeria between July 2007 and July 2012. Information was retrieved from patients’ clinical notes and analyzed using statistical package for social sciences (SPSS) version 16.0.

**Results:**

Eighty-four patients were treated during the period of study, their ages ranged between 19 and 89 years with the mean age of 52.3 years. The mean duration of symptoms before presentation was 3 years and 1 month. Inflammation resulting from sexually transmitted infection was the commonest etiology and more than 50% of the patients presented with complications. Sixteen patients (19.1%) received no treatment due to lack of fund. More than 90% were dependent, unemployed or underemployed. Single stage reconstruction by urethral substitution was the commonest form of repair with the restenosis rate of 4.4%.

**Conclusions:**

Prevalent socio-cultural and economic situation in south western Nigeria have added some peculiar challenges to the management of urethral stricture in the region.

## 1. Background

Urethral stricture is defined as narrowing of the urethra caused by scarring of the urethral epithelium and the peri-urethral spongy tissue. Although it is one of the oldest known urological diseases, it constitutes a significant workload for urologists globally with attendant high morbidity (1, 2). Its management has continued to present a formidable and enormous challenge to urologic surgeons (3). With improvement in living standards and understanding the pathology of the disease, coupled with recent advances in imaging, endoscopy and surgical practices, remarkable improvements have been achieved in the management of urethral stricture with evolution of several modalities of treatment (4).

In Africa, the changes in the natural history of urethral stricture may not be comparable with what is seen in the developed world. Poor awareness, late presentation, and persistence of rare complications have beguiled the management of urethral stricture in most African nations (2).

## 2. Objectives

We conducted this study to document the challenges faced by urologists treating urethral stricture in Osogbo, south western Nigeria, and to report the effect of socio-cultural and economic situations of such community on the management of urethral stricture.

## 3. Patients and Methods

We performed a retrospective review of all cases of urethral stricture disease managed over a 5 year period (July 2007–July 2012) in the urology unit of Ladoke Akintola University of Technology, Nigeria, a tertiary health institution in Osogbo, a semiurban city in rain forest belt of south western part of Nigeria and the capital city of Osun state. Most dwellers are traders, artisans, farmers, and educated salary earners in public and private services. The hospital serves as the referral center for urological diseases from hospitals in Osogbo and the neighboring rural communities.

After clearance from the ethical committee of the hospital, all patients presenting with urethral stricture disease confirmed by urethrography and/or urethrocystoscopy during the period of the study were included in the study. For this study, we defined urethral stricture as narrowing of the urethra confirmed by urethrogram and severe enough to cause symptoms and/or complications for the patient. We excluded patients with symptoms not confirmed by investigation, patients who had symptoms from other lesions different from urethral stricture, and patients with incomplete records because of failure to complete treatment in our unit. Information on age, occupation, duration of symptoms, aetiology, treatment before presentation, complications, treatment in the unit, and causes of primary, secondary and/or tertiary delays in receiving treatment were entered on a preformed proforma. Investigation modalities used for confirmation of diagnosis included urethrography and urehtrocystoscopy. Data collected was entered intocomputer and analyzed with statistical package for social sciences (SPSS) version 16.0 using simple descriptive statistical analysis.

## 4. Results

Eighty-four patients with urethral stricture presented in our unit during the period of the study and they were all included in the study. Their ages ranged between 19 and 89 years with the mean age of 52.3 years. Duration of symptoms before presentation ranged between 2 months and 8 years with the mean duration of 3 years and 1 month. All the patients had lower urinary tract symptoms (LUTS) at presentation, and 73 (86.9%) had various forms of complications at presentation as shown in Table 1. 

**Table 1.** Complications at Presentation

Complications	Frequency	Percentage
**Urethrocutaneous fistula**	5	6.0
**Urine retention**	44	52.4
**Bladder/urethral calculi**	8	9.5
**Chronic renal impairment**	5	6.0
**Significant urinary tract infection**	51	60.7

**Figure 1.**
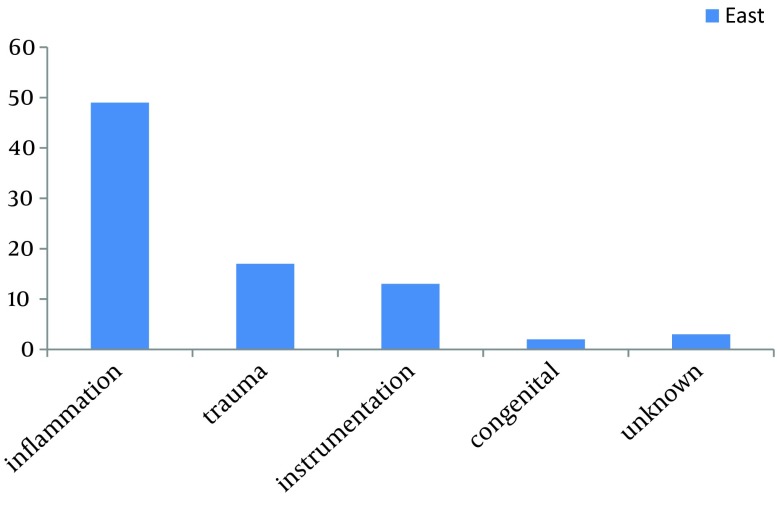
Aetiology

Inflammation from sexually transmitted infection was the commonest etiology (58.3%) as shown in Figure 1. Sixty-nine (82.1%) patients were either unemployed, underemployed or dependent. 

**Figure 2.**
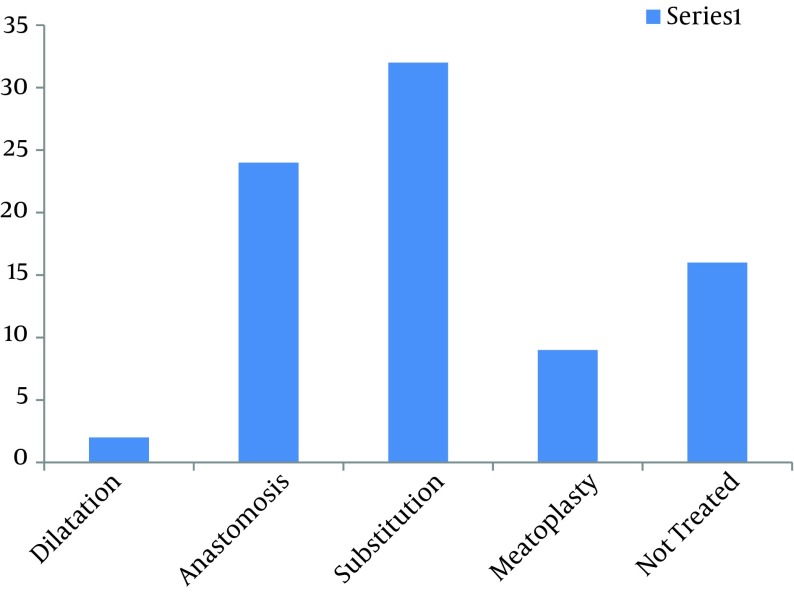
Treatment Offered

Various treatments performed before presentations are shown in Table 2, and 4 patients (4.8%) were wrongly diagnosed and had undergone surgical operation before presentation. We confirmed the diagnosis with urethrocystoscopy in 6 patients (7.14%), but all the patients were evaluated with urethrography. Location of the stricture along the urethra is shown in Table 3 with the commonest site being the bulbar segment in 37 patients (44.1%). The duration between presentation to our unit and treatment ranged between 1 and 23 months with the mean duration of 5 months. The major cause of delay in getting treatment was lack of funds to pay for treatment; others included delay in getting investigations performed due to large patients’ load with limited facility, industrial actions (strike) by health care providers, and a high load of patients requiring surgical treatment with limited operating rooms causing backlogs of patients awaiting surgery. Of these patients, only 68 (81.0%) were treated during the period of the study, and 16 (19.0%) remained untreated due to financial constraints. Treatment offered is shown in Figure 2 with substitution urethroplasty being the commonest. Postoperative complications were observed in 9 patients giving a complication rate of 13.2%. Various complications resulting from the treatment are shown in Table 4. 

**Table 2.** Treatments Before Presentation

Treatment	Frequency	Percentage
**Suprapubic diversion**	61	72.6
**Urethral dilatation**	39	46.4
**Internal urethrotomy**	3	3.6
**Open prostatectomy**	4	4.8

**Table 3.** Site of Stricture

Stricture site	Frequency	Percentage
**Penile**	11	13.1
**Peno-bulbar**	9	10.7
**Bulbar**	37	44.1
**Bulbo-membranous**	16	19.5
**External meatus**	11	13.1

**Table 4.** Complications of Treatment

Treatment	Frequency	Percentage
**Wound infection**	7	10.3
**Urethrocutaneous fistula**	4	5.9
**Flap necrosis**	3	4.4
**Donor site (penile wound) infection **	3	4.4
**Restenosis**	3	4.4

The duration of hospital stay was between 15 and 38 days with the mean hospital stay of 20.33 days. The duration of follow up ranged between 8 and 37 weeks with the mean duration of 21 weeks.

## 5. Discussion

Urethral stricture is one of the oldest and commonest urological problems treated by urologists; it has remained a major challenge till date (2). Prevalence of urethral stricture among people of poor socio-economic status is noted in our environment: four of five of our patients were either dependent, unemployed or under employed, while one of five remained untreated due to lack of fund. This is not different from earlier reports from other parts of Africa due to the prevalence of poverty and unemployment in this region (2, 5, 6). The average cost of urethral reconstruction for stricture in our hospitals was between 800 and 1000 USD. This is expensive for most people living in this environment where health insurance covers only a minute percentage of the population (7). This translates to each patient funding his treatment directly and it explains why lack of fund is the major reason for delay or nontreatment among these patients.

In most developed nations, inflammation is becoming relatively uncommon among causes of urethral stricture with trauma taking the lead (8). However, inflammation has remained the commonest aetiology in our environment because of the high prevalence of gonorrhoea, and other sexually transmitted infections (9). Stricture resulting from trauma is short in length and the fibrosis is limited to the site of injury making the reconstruction relatively nontasking. However, stricture from inflammation is usually associated with dense and relatively extensive peri-urethral fibrosis, usually involving a long urethral segment and may be found in multiple sites (9). Infection is most commonly due to Neisseria gonorrhea and infrequently to Chlamydia and other nongonococcal organisms which are usually transmitted sexually (10). In most African communities, it is culturally shameful for men and women to discuss diseases affecting their perineum and external genitalia because of societal stigmatization (11). Poverty, ignorance, and superstitious beliefs in diabolical causes of illness make these patients vulnerable. Most of them visit spiritualists and herbalists who claim ancestral and supernaturally inherited powers used to perform healing (12, 13). Therefore, late presentation with complications is the rule after several visits to spiritualists and traditional healers. One is therefore not surprised with the delay in presentation and the complications recorded in these patients. Until there is increased awareness and prompt treatment of sexually transmitted infections, urologists in sub Saharan Africa would continue to treat more of complex and complicated cases of urethral stricture diseases (14, 15).

Complications of long standing urethral stricture disease are noted in many of our patients; every other patient came with urine retention and one of every eight patients with bladder/urethral calculi. Chronic renal impairment and perineal fistulae resulting from urethral stricture are now uncommon in many parts of the world (14). They increase the challenges posed to the managing urologist with increased pre and post treatment morbidity, delay in time to definitive treatment, needs for complex reconstructive procedures and significant increase in the overall cost of treatment in a society where fund is a major limitation to treatment (11, 16).

Paucity of urologists has contributed immensely to these challenges with few available ones concentrated in major tertiary hospitals in urban centers (6). This is why most of our patients were managed by nonspecialists before presentation. Some were wrongly diagnosed and treated with undue delay in accessing proper treatment, waste of available meager funds, and worsening of patients problem before presentation in a specialized unit (16-18).

Substitution reconstruction is the most common repair in our patients because most of the strictures were complex and not amenable to simple treatments like dilatation, or resection and anastomosis. Post inflammatory strictures involving more than 2 cm urethral segment with extensive peri-urethral fibrosis and occasional peri-urethral abscess or fistula may not leave many options to the managing surgeon (19). Where there is severe spongio-fibrosis, urethro-cutaneous fistula or peri-urethral abscess, we raised a fascio-cutaneous flap from the distal penile skin on a vascularised pedicle. This is sutured to the native urethra as an ‘onlay flap’ to complete urethral circumference. This tissue is considered reliable because it carries its own blood supply; it does not depend on the condition of the recipient site which may be precarious and unable to support a graft (20). Though no study has yet been able to establish the superiority of penile skin flap over buccal mucosal graft but a vascularised flap is still preferred by some urologists where the conditions of the recipient site may interfere with ‘graft survival’ (21). However, where there was minimal fibrosis without peri-urethral abscess or fistula, we used buccal mucosa tissue raised as a graft, and used as dorsal onlay flap to reduce the chances of urethral diverticulum.

Follow up clinic attendance was not encouraging. Most patients would stop attending follow up clinics unless they had some postoperative problems. This does not allow the managing surgeon to evaluate the long term results of the treatments received.

Conclusion: prevalent socio-economic situation and cultural beliefs in south western Nigeria have added more challenges to the management of urethral stricture in the region. The situation may not be different in most developing nations. Urologists intending to practice in such communities, and those currently practising must harm themselves with various modalities of treatment to handle complex and complicated cases of urethral stricture diseases. 
